# Real-World Effectiveness of Benralizumab in Severe Eosinophilic Asthma: A One-Year Single-Center Retrospective Study

**DOI:** 10.3390/jcm15218360

**Published:** 2026-10-27

**Authors:** Dane Ediger, Raziye Tülümen Öztürk, Aylin Arslan, İsmail Emre Deniz

**Affiliations:** 1 Department of Pulmonary Diseases, Division of Allergy and Immunology, Faculty of Medicine, Bursa Uludag University, 16059 Bursa, Türkiye; rtulumenozturk@uludag.edu.tr (R.T.Ö.); aylinalancayir@gmail.com (A.A.); 2 Department of Biostatistics, Institute of Health Sciences, Bursa Uludag University, 16059 Bursa, Türkiye

**Keywords:** severe eosinophilic asthma, benralizumab, biologic therapy, real-world evidence, treatment response, super-responder

## Abstract

**Background/Objectives**: Benralizumab is effective in severe eosinophilic asthma, but real-world evidence on response characteristics and outcomes after switching from other biologics remains limited. This study evaluated one-year clinical, laboratory, and functional outcomes and factors associated with response. **Methods**: This single-centre retrospective observational study included 53 adults with severe eosinophilic asthma who reached the week 16 assessment; 40 continued treatment and completed 12 months. Outcomes were assessed at baseline, week 16, and month 12. **Results**: At week 16, 40 patients (75.5%) were responders and 13 (24.5%) were non-responders. Among 12-month completers, exacerbations, asthma-related hospitalisations, and systemic corticosteroid courses decreased significantly. Asthma Control Test scores and pulmonary function improved significantly, while blood eosinophil counts decreased markedly. Responders were older and more frequently atopic than non-responders. Improvement did not differ significantly among biologic-naïve patients and those switched from omalizumab or mepolizumab. Five patients (12.5%) met the super-responder criteria. **Conclusions**: Among patients who completed 12 months, benralizumab treatment was associated with improved asthma control and pulmonary function and fewer exacerbations and systemic corticosteroid courses. Changes did not differ significantly according to previous biologic treatment.

## 1. Introduction

Asthma is a heterogeneous disease characterised by variable respiratory symptoms, expiratory airflow limitation, and chronic airway inflammation [1]. Severe asthma remains uncontrolled despite confirmation of the diagnosis, optimisation of adherence and inhaler technique, management of contributing factors and comorbidities, and high-dose inhaled corticosteroids (ICS) plus a second controller, or worsens when this treatment is reduced. Approximately 3.7% of patients with asthma are estimated to meet the criteria for severe asthma [1,2]. These patients have a significant symptom and exacerbation burden. Serious complications of repeated or continuous use of systemic corticosteroids include obesity, diabetes, osteoporosis, cardiovascular disease, cataracts and adrenal suppression.

In severe asthma with predominant type 2 inflammation, biologic therapies are essential treatment options because they target the inflammatory pathways that underlie the disease. GINA recommends the consideration of biologic therapy in patients with severe asthma uncontrolled on high-dose ICS–long-acting beta_2_-agonist therapy and with appropriate features of type 2 inflammation [1]. Benralizumab is a humanized, afucosylated IgG1κ monoclonal antibody that binds to the alpha subunit of the interleukin-5 receptor. Antibody-dependent cell-mediated cytotoxicity induces rapid depletion of eosinophils [3].

Benralizumab decreased exacerbations and enhanced lung function in individuals with severe eosinophilic asthma, according to the randomized, double-blind, phase III, placebo-controlled CALIMA and SIROCCO studies [4,5]. Benralizumab’s ability to spare oral corticosteroids in patients with oral corticosteroid-dependent severe asthma was shown by the ZONDA trial [6], and an integrated analysis including the MELTEMI extension study supported long-term safety and sustained reductions in exacerbations for up to five years [7]. Similar outcomes have been seen in real-world studies in more heterogeneous patient populations than in clinical trials [8,9,10,11]. Nevertheless, real-world evidence regarding patient characteristics associated with response to benralizumab and the influence of previous biologic therapy on clinical outcomes remains limited.

Previous real-world studies, including reports from Türkiye [12,13], have documented improvements with benralizumab. We evaluated week 16 clinical response in a Turkish severe asthma cohort and 12-month outcomes among patients who continued treatment, with particular attention to previous biologic treatment and super-response.

## 2. Materials and Methods

### 2.1. Study Design and Patient Population

This single-centre, retrospective observational study screened patients aged ≥18 years who were followed with a diagnosis of severe eosinophilic asthma at the Pulmonology Allergy Outpatient Clinic of Bursa Uludağ University Faculty of Medicine Hospital and had a benralizumab treatment authorisation record between November 2023 and March 2025. Demographic, clinical, laboratory, and pulmonary function data were retrospectively obtained from electronic medical records.

A total of 75 patients were assessed for eligibility. Seven patients with insufficient clinical or follow-up data, six who did not complete 12 months of follow-up, three whose treatment was discontinued before the scheduled week 16 assessment, two who had prolonged interruptions in treatment, two with a diagnosis or suspected diagnosis of eosinophilic granulomatosis with polyangiitis, one who received benralizumab within a clinical trial, and one who did not actually initiate benralizumab treatment were excluded. The study cohort comprised 53 patients who reached the week 16 response assessment. Patients whose treatment was discontinued at week 16 because of non-response were included in the response analysis, whereas the 12-month effectiveness and super-response analyses were conducted among the 40 patients who continued treatment and completed one year of follow-up.

Patients diagnosed with severe eosinophilic asthma according to the Global Initiative for Asthma (GINA) criteria and receiving at least high-dose inhaled corticosteroid (ICS) plus long-acting beta_2_-agonist (LABA) therapy before benralizumab initiation were included. The eosinophilic asthma phenotype was defined as a peripheral blood eosinophil count of ≥300 cells/µL or ≥150 cells/µL in patients who had received regular oral corticosteroid therapy during the preceding year or at the time of assessment [1]. For patients previously treated with another biologic, earlier clinical and laboratory records were also considered when determining the eosinophilic phenotype.

Benralizumab was administered subcutaneously at a dose of 30 mg every four weeks for the first three doses and every eight weeks thereafter.

The study followed the standards of the Declaration of Helsinki and was approved by the Clinical Research Ethics Committee at Bursa Uludağ University Faculty of Medicine (approval date: 6 May 2026; decision no: 2026/254/9-9).

### 2.2. Recorded Variables and Definitions

Age at benralizumab initiation, sex, age at asthma onset, asthma duration, body mass index, smoking history, smoking exposure, comorbidities, atopic status, and previous biologic treatment history were recorded. The presence of rhinitis symptoms, nasal polyps, gastro-oesophageal reflux disease, anxiety/depression, osteoporosis, coronary artery disease, diabetes mellitus, obstructive sleep apnoea syndrome, and non-steroidal anti-inflammatory drug-exacerbated respiratory disease was determined from clinical assessments and physician diagnoses documented in the electronic medical records.

Atopy was assessed using skin prick testing and/or serum-specific IgE measurements. A skin prick test was considered positive when the wheal diameter was at least 3 mm greater than that of the negative control after 15–20 min. A serum-specific IgE level of ≥0.35 kUA/L was considered positive. Allergen sensitisation was classified as perennial or seasonal. The presence of rhinitis symptoms was retrospectively determined based on the documentation of compatible nasal symptoms in the electronic medical records.

According to previous biologic treatment history, patients were classified as biologic-naïve, switched from omalizumab to benralizumab, or switched from mepolizumab to benralizumab.

Asthma control was assessed using the Asthma Control Test (ACT). A clinically significant asthma exacerbation was defined as an event involving worsening asthma symptoms that required the initiation of systemic corticosteroids and/or an emergency department visit or hospitalisation.

### 2.3. Assessment of Treatment Effectiveness

Treatment effectiveness was assessed at baseline, week 16, and month 12. To determine baseline disease burden, the numbers of asthma exacerbations, asthma-related hospitalisations, and systemic corticosteroid courses during the 12 months preceding benralizumab initiation were recorded. Among patients who continued treatment for 12 months, these clinical events were compared with those occurring during the first 12 months of benralizumab treatment.

Peripheral blood eosinophil counts, ACT scores, and pulmonary function parameters were recorded at benralizumab initiation, week 16, and month 12. Pulmonary function was assessed using forced expiratory volume in one second (FEV_1_), expressed in litres and as a percentage of the predicted value. Spirometry was performed using regularly calibrated devices in accordance with American Thoracic Society/European Respiratory Society standards [14]. Whenever possible, measurements were obtained during periods of clinical stability and under routine follow-up conditions.

Baseline-to-week 16 comparisons were performed among the 53 patients who reached the week 16 assessment, whereas baseline-to-month 12 comparisons were performed among the 40 patients who continued treatment and completed 12 months of follow-up. For analyses of exacerbations, hospitalisations, and systemic corticosteroid courses, the 12-month period before treatment was compared with the first 12 months of benralizumab treatment.

### 2.4. Classification of Treatment Response

Treatment response at week 16 was determined through routine clinical assessment after the first four doses of benralizumab. This assessment jointly considered asthma exacerbation burden, systemic corticosteroid requirement, asthma control, pulmonary function, and the patient’s overall clinical status. Patients for whom the treating physician decided to continue benralizumab were classified as “responders”, whereas those whose treatment was discontinued because of clinical non-response were classified as “non-responders”.

At month 12, super-response was assessed using predefined major and minor criteria. The absence of exacerbations during the 12-month treatment period, a large improvement in asthma control (an increase in ACT score of ≥6 points, corresponding to at least twice the minimal clinically important difference), and discontinuation of maintenance oral corticosteroid therapy used at baseline were considered major criteria. A ≥75% reduction in the number of exacerbations relative to baseline, achievement of well-controlled asthma (ACT score ≥ 20), and an increase of ≥500 mL in FEV_1_ relative to baseline were defined as minor criteria. Patients who met at least two major criteria and at least three criteria overall were classified as “super-responders”. Patients who responded to treatment but did not meet these criteria were classified as “non-super-responders” [15].

### 2.5. Statistical Analysis

The normality of continuous variables was assessed using the Shapiro–Wilk test. Normally distributed continuous variables are presented as mean ± standard deviation, non-normally distributed variables as median (Q1–Q3), and categorical variables as number and percentage. Q1 and Q3 denote the 25th and 75th percentiles, respectively.

Two independent groups were compared using the independent-samples *t*-test or Mann–Whitney U test, depending on the distribution of the variables. Comparisons among more than two independent groups were performed using one-way analysis of variance or the Kruskal–Wallis test.

Measurements obtained from the same patients at two time points were compared using the paired-samples *t*-test or Wilcoxon signed-rank test. Categorical variables were compared using Pearson’s chi-square test, Fisher’s exact test, or the Fisher–Freeman–Halton test, as appropriate.

For between-group comparisons according to previous biologic treatment history, change from baseline scores were calculated as the follow-up value minus the baseline value. Each paired analysis included patients with available data at both relevant time points.

Effect sizes were reported as Cohen’s d for paired-samples t-tests and the rank-biserial correlation coefficient for Wilcoxon signed-rank tests. Effect size estimates were interpreted alongside the direction and magnitude of the observed changes and their corresponding *p* values. For interpretation, absolute Cohen’s d values of 0.20 to <0.50, 0.50 to <0.80, and ≥0.80 were classified as small, medium, and large, respectively; absolute rank-biserial correlation coefficients of 0.10 to <0.30, 0.30 to <0.50, and ≥0.50 were classified as small, medium, and large, respectively. These categories were used as descriptive conventions and do not, by themselves, establish clinical importance.

As an exploratory sensitivity analysis, baseline blood eosinophil counts were compared between week 16 responders and non-responders after excluding patients previously treated with mepolizumab. Comparisons of baseline characteristics between responders and non-responders were exploratory, and no correction for multiple comparisons was applied in these analyses. All tests were two-sided, and *p* < 0.05 was considered statistically significant. Statistical analyses were performed using IBM SPSS Statistics, version 25 (IBM Corp., Armonk, NY, USA).

## 3. Results

### 3.1. Baseline Demographic, Clinical, and Laboratory Characteristics of the Study Population

**Figure 1:**
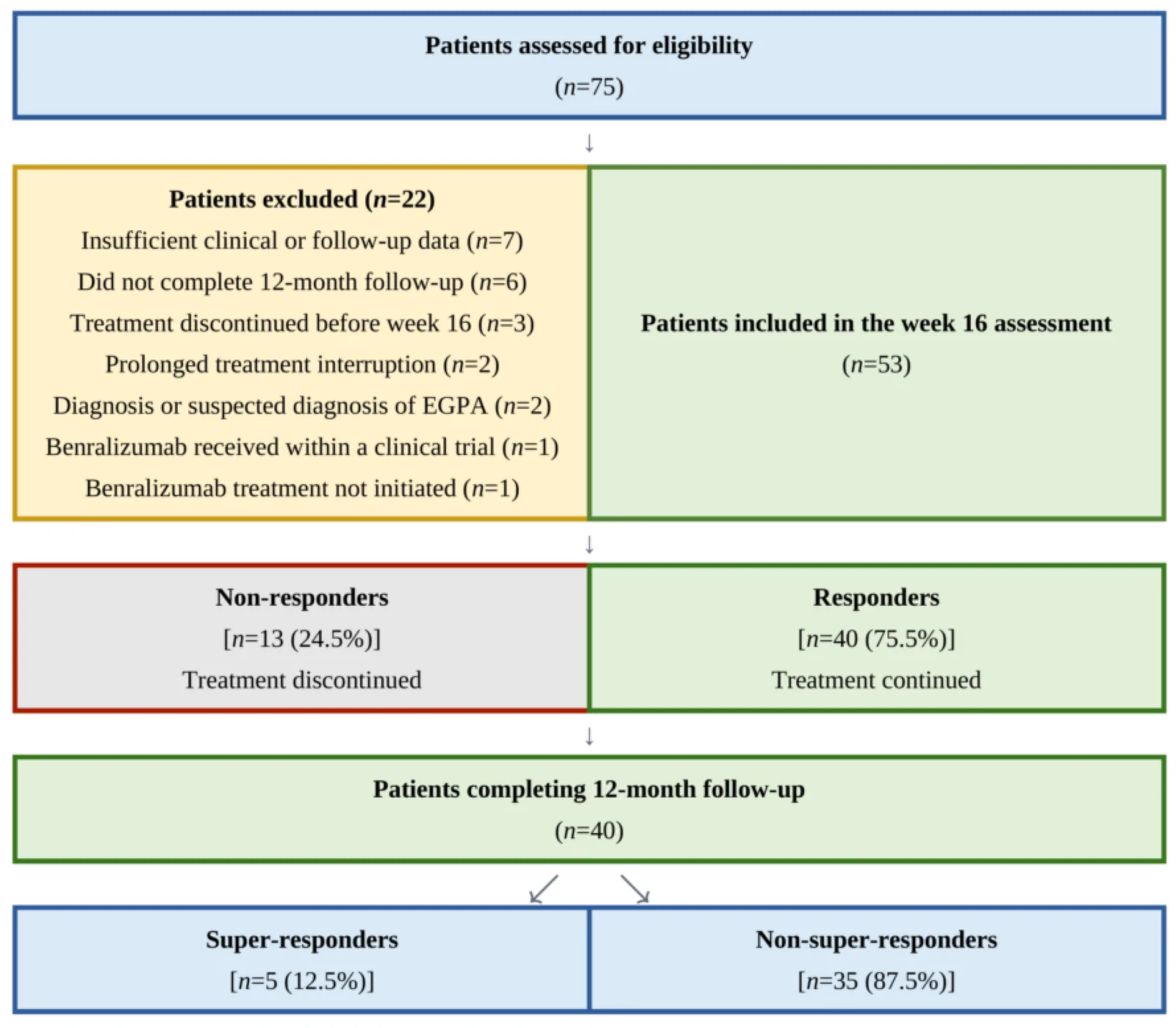
Flow diagram of patient selection and treatment response during follow-up.

A total of 75 patients were assessed for eligibility between November 2023 and March 2025. Of the 53 patients included in the analysis, treatment was discontinued in 13 (24.5%) because of non-response following the week 16 response assessment. The remaining 40 patients (75.5%), who responded to treatment at week 16, completed 12 months of follow-up; 35 (87.5%) were classified as non-super-responders and five (12.5%) as super-responders. The patient selection and treatment-response process is shown in Figure 1.

The mean age of the patients was 54.68 ± 11.07 years (range, 31–77 years), and 86.8% were female. The mean age at asthma onset was 37.09 ± 12.03 years, the median duration of asthma was 16 years, and the median body mass index was 30 kg/m^2^. Overall, 62.3% of the patients had never smoked, and none were current smokers.

Rhinitis symptoms were present in 94.3% of the patients, atopy in 64.2%, and nasal polyps in 15.1%. The median baseline total IgE level was 112 IU/mL, and the median peripheral blood eosinophil count was 329 cells/µL. The mean FEV_1_ was 1.78 ± 0.67 L, the mean FEV_1_ percentage of predicted was 67.72 ± 21.17%, and the mean ACT score was 13.45 ± 4.12.

During the 12 months preceding benralizumab initiation, the median numbers of asthma exacerbations, systemic corticosteroid courses, and hospitalisations were 3, 2, and 0, respectively. Of the patients, 20.8% were biologic-naïve, whereas 22.6% had switched from omalizumab and 56.6% from mepolizumab to benralizumab. The baseline demographic, clinical, laboratory, and functional characteristics of the study population are presented in Table 1.

### 3.2. Clinical and Functional Effects of Benralizumab Treatment

Among the 40 patients who completed 12 months of benralizumab treatment, the annual number of asthma exacerbations decreased from a median (Q1–Q3) of 3 (2–4) before treatment to 0 (0–1) during treatment (*p* < 0.001). Asthma-related hospitalisation counts also decreased significantly, although the median (Q1–Q3) was 0 (0–0) in both periods (*p* = 0.015). The number of patients with at least one asthma-related hospitalisation decreased from 7 (17.5%) to 1 (2.5%). Systemic corticosteroid courses decreased from 2 (1–3) to 0 (0–0) per year (*p* < 0.001) (Table 2).

Peripheral blood eosinophil counts decreased from a median (Q1–Q3) of 329 (71.5–534) to 1 (0–4) cells/µL at week 16 among 53 patients and from 288.5 (64.75–510.75) to 2.5 (1–4) cells/µL at month 12 among 40 patients (*p* < 0.001 for both comparisons). Mean ACT scores increased by 3.81 points at week 16 and 7.48 points at month 12 (*p* < 0.001 for both comparisons). Both mean changes exceeded the 3-point minimal clinically important difference for the ACT.

Pulmonary function also improved significantly. Compared with baseline, mean FEV_1_ increased by 0.20 L at week 16 and by 0.30 L at month 12 (*p* < 0.001 for both comparisons). Similarly, mean FEV_1_ percentage of predicted increased from baseline by 7.47 percentage points at week 16 and by 10.60 percentage points at month 12 (*p* < 0.001 for both comparisons) (Table 2). At the month 12 assessment, improvements in laboratory findings, asthma control, and pulmonary function were maintained among patients who continued treatment.

Effect sizes are presented in Table 2. According to the specified thresholds, reductions in annual exacerbations and systemic corticosteroid courses showed large effects, whereas the reduction in asthma-related hospitalisations showed a medium effect. Reductions in blood eosinophil counts and improvements in ACT scores showed large effects at both assessments. The effect on FEV_1_ (L) was small at week 16 and medium at month 12; effects on FEV_1_ (% predicted) were medium at both assessments.

### 3.3. Evaluation According to Treatment Response

At the week 16 assessment, 40 of the 53 patients (75.5%) were classified as responders to benralizumab, whereas 13 (24.5%) were classified as non-responders, and treatment was discontinued in these patients. Responders were older than non-responders (56.98 ± 10.16 vs. 47.62 ± 11.13 years; *p* = 0.007). Atopy was also more frequent among responders [29/40 (72.5%) vs. 5/13 (38.5%); *p* = 0.044]. No significant between-group differences were identified in the other baseline demographic and clinical characteristics, previous biologic treatment history, or indicators of disease burden during the 12 months preceding treatment (*p* > 0.05 for all comparisons) (Table 3). After exclusion of patients previously treated with mepolizumab, baseline eosinophil counts did not differ significantly between responders (*n* = 16; median, 413.5 cells/µL) and non-responders (*n* = 7; median, 427 cells/µL; *p* = 0.867). Pruritus was reported in two patients whose treatment was discontinued after the week 16 assessment. Both patients also had an inadequate clinical response.

The 40 patients who responded to treatment at week 16 continued benralizumab for 12 months and were subsequently evaluated according to the degree of response. Of these patients, 35 (87.5%) were classified as non-super-responders and five (12.5%) as super-responders. No patients were lost to follow-up during this period, and all completed the 12-month assessment. No statistically significant differences were identified between non-super-responders and super-responders in baseline demographic, clinical, laboratory, or functional characteristics or previous biologic treatment history (*p* > 0.05 for all comparisons) (Table 4).

### 3.4. Evaluation According to Previous Biologic Treatment History

Among biologic-naïve patients, the annual numbers of asthma exacerbations and systemic corticosteroid courses during the first year of benralizumab treatment decreased significantly compared with the 12 months before treatment (*p* = 0.017 for both comparisons), whereas the change in the number of asthma-related hospitalisations was not significant (*p* = 0.180). Peripheral blood eosinophil counts decreased significantly from baseline at both week 16 and year 1 (*p* = 0.003 and *p* = 0.012, respectively), whereas ACT scores (*p* = 0.002 and *p* < 0.001, respectively), FEV_1_ (*p* = 0.010 and *p* = 0.013, respectively), and FEV_1_ percentage of predicted (*p* = 0.020 and *p* = 0.035, respectively) increased significantly at both assessment time points.

When biologic-naïve patients were compared with those who switched from omalizumab or mepolizumab to benralizumab, no significant between-group differences were observed in the magnitude of changes in ACT scores, peripheral blood eosinophil counts, FEV_1_, or FEV_1_ percentage of predicted at week 16. Similarly, at year 1, changes in the annual numbers of asthma exacerbations, asthma-related hospitalisations, and systemic corticosteroid courses, as well as changes in ACT scores, peripheral blood eosinophil counts, and pulmonary function, did not differ significantly among the groups (*p* > 0.05 for all comparisons) (Table 5).

## 4. Discussion

In this retrospective real-world study, significant improvements in asthma control and pulmonary function were observed in patients with severe eosinophilic asthma as early as week 16 of benralizumab treatment and were maintained through the first year. During the first year of treatment, the numbers of asthma exacerbations, asthma-related hospitalisations, and systemic corticosteroid courses decreased significantly compared with the 12 months preceding treatment. These findings are consistent with those of the randomised, double-blind, placebo-controlled phase III CALIMA and SIROCCO trials, which demonstrated that benralizumab reduced asthma exacerbation rates [4,5]. Furthermore, the marked reduction in peripheral blood eosinophil counts observed at week 16 and maintained through the first year is consistent with the mechanism of action of benralizumab, which targets IL-5Rα and induces eosinophil depletion through antibody-dependent cell-mediated cytotoxicity [5].

The clinical effectiveness of benralizumab has also been demonstrated in real-world cohorts with diverse patient characteristics. In the multinational retrospective XALOC-1 programme, benralizumab treatment was associated with improvements in asthma control and pulmonary function, as well as a reduction in maintenance oral corticosteroid use [16]. Similarly, the BREEZE study, which included patients from Central and Eastern Europe and the Baltic countries, reported reductions in exacerbations and oral corticosteroid use alongside improvements in asthma control and lung function [17]. Real-world data from Türkiye have also shown at two centres that reductions in exacerbations were maintained through week 52 [12]. These findings are broadly consistent with the clinical and functional improvements observed at year 1 in our study. Another study from Türkiye reported improvements in asthma control, exacerbations, and lung function over approximately 32 weeks in 24 biologic-naïve patients treated with benralizumab [13]. Our cohort also included patients who had switched from omalizumab or mepolizumab and evaluated 12-month outcomes among treatment continuers. Differences in patient selection, response definitions, and follow-up periods should be considered when comparing these studies.

Asthma control improved at both assessments. Among the 53 patients assessed at week 16, mean ACT scores increased from 13.45 ± 4.12 to 17.26 ± 4.87. Among the 40 patients who completed 12 months, scores increased from 13.83 ± 4.22 at baseline to 21.30 ± 3.39 at month 12. The mean changes of 3.81 and 7.48 points, respectively, exceeded the 3-point minimal clinically important difference for the ACT [18]. Sözener et al. reported clinically meaningful improvements in ACT scores at weeks 8, 24, and 52 in a real-world study from Türkiye [12]. In BREEZE, the median increase was 9 points at weeks 16 and 24 and 9.5 points at week 48 [17]. Improvements in asthma control were also reported in XALOC-1, ORBE II, and other real-world cohorts [16,19,20,21].

Improvements in pulmonary function were evident at week 16 and persisted through the first year. Compared with baseline, mean FEV_1_ increased by 0.20 L at week 16 and by 0.30 L at year 1. The corresponding mean increases in FEV_1_ percentage of predicted were 7.47 and 10.60 percentage points, respectively (*p* < 0.001 for all comparisons). The magnitude of FEV_1_ improvement has varied across real-world studies. In the XALOC-1 programme, the least-squares mean increase in pre-bronchodilator FEV_1_ was 270 mL at week 48 [16], and the increase reached approximately 300 mL at week 48 in its Italian component, the ANANKE study [22]. Pelaia et al. reported increases of 390 and 530 mL at months 1 and 6, respectively [23]. Other real-world studies have similarly demonstrated variability in FEV_1_ response across cohorts [20,21,24]. These differences may be related to variations in baseline disease burden and phenotypic characteristics among study populations.

The mean age of our cohort (54.68 years; range, 31–77 years) was comparable to that reported in several real-world benralizumab cohorts, including BREEZE (56 years) [17], ORBE II (56.4 years) [19], and XALOC-1 (55.4 years) [16]. A two-centre study from Türkiye reported a somewhat lower mean age of approximately 50.1 years [12]. In the response-based comparison, responders were older than non-responders. However, a recent systematic review and meta-analysis investigating predictors of response to biologic therapy found no significant association between age and clinical response specifically to benralizumab, and the certainty of the evidence was considered very low [25]. Therefore, the association between age and treatment response observed in our study should be interpreted cautiously and requires confirmation in larger patient populations. In the same meta-analysis, a high blood eosinophil count (≥300 cells/µL) and a high fractional exhaled nitric oxide level (>40 ppb) were among the biomarkers most strongly associated with treatment response [25]. In our study, the peripheral blood eosinophil count at benralizumab initiation was not significantly associated with treatment response. Interpretation of this finding is complicated by the fact that most patients had previously received biologic therapy. In particular, among patients who switched from mepolizumab to benralizumab, the eosinophil count at benralizumab initiation may have been suppressed by previous anti-IL-5 treatment and may therefore not accurately reflect the pretreatment burden of eosinophilic inflammation. Consequently, the absence of an association between baseline eosinophil count and response in our cohort should not be interpreted as evidence that eosinophils have no predictive value for treatment response. The absence of a significant difference in baseline eosinophil counts persisted in an exploratory analysis excluding mepolizumab-experienced patients, although the small number of remaining patients limits interpretation of this analysis.

Atopy was more frequent among responders to benralizumab than among non-responders (72.5% vs. 38.5%; *p* = 0.044). No statistically significant associations were observed between treatment response and baseline total IgE levels or perennial or seasonal allergen sensitisation. Evidence regarding the relationship between atopy and response to benralizumab remains inconsistent. In a multicentre real-world study of 111 patients with severe eosinophilic asthma, Pelaia et al. found benralizumab to be effective in both skin prick test (SPT)-positive and SPT-negative patients. However, reductions in exacerbations and short-acting beta_2_-agonist use and increases in ACT scores were more pronounced among SPT-positive patients, whereas baseline total IgE levels were not associated with treatment outcomes [23]. Similarly, in the long-term real-world study by Sposato et al., patients with an allergic phenotype showed greater improvements in ACT scores and FEV_1_ and a greater reduction in SABA use than those with a non-allergic phenotype. Among patients receiving maintenance oral corticosteroids, the proportion achieving a dose reduction of at least 50% was also higher in the allergic group [26].

Conversely, a pooled analysis of the SIROCCO and CALIMA trials demonstrated that the effects of benralizumab on exacerbations and lung function were independent of atopic status and serum IgE levels [27]. The ANANKE study likewise reported that the effectiveness of benralizumab was unaffected by allergic status [22]. In a cluster analysis examining benralizumab response phenotypes, the exacerbation-free response rate was higher in the group with both high IgE and high blood eosinophil levels; however, an independent role of atopy in treatment response could not be demonstrated [28]. The higher prevalence of atopy among responders in our study is consistent with some real-world evidence. Nevertheless, the small number of non-responders and the lack of statistically significant associations with total IgE levels or allergen sensitisation warrant cautious interpretation. Larger studies are needed to clarify the role of atopy in the response to benralizumab.

We found no significant differences between biologic-naïve patients and those who switched from omalizumab or mepolizumab to benralizumab in the magnitude of changes in exacerbations, systemic corticosteroid use, ACT scores, peripheral blood eosinophil counts, or pulmonary function. Real-world studies reporting favourable outcomes after switching to benralizumab in patients inadequately controlled with previous biologic therapy support our findings. Among patients with severe eosinophilic asthma who remained uncontrolled despite omalizumab treatment, switching to benralizumab was associated with improvements in asthma control, exacerbation frequency, lung function, and eosinophil counts [29]. Similarly, switching to benralizumab in patients with an inadequate response to mepolizumab or reslizumab has been shown to improve asthma control and FEV_1_ and reduce oral corticosteroid requirements [30]. The targeting of IL-5Rα by benralizumab and its ability to induce rapid and profound eosinophil depletion through antibody-dependent cell-mediated cytotoxicity may explain the additional benefit observed after switching in some patients whose disease was not adequately controlled with anti-IL-5 therapy [31]. In a post hoc analysis from the ANANKE programme, Caruso et al. reported reductions in the annualised exacerbation rate of 93.6% in biologic-naïve patients and 91.9% in biologic-experienced patients; ACT scores and lung function also improved in both groups [32]. A subsequent analysis from the same programme reported sustained clinical benefits in both groups up to 96 weeks [22]. Thus, substantial improvement after switching is possible, but our small subgroups, particularly at month 12, limit the ability to detect between-group differences. The absence of a statistically significant difference does not establish equivalent effectiveness.

Of the 40 patients who completed 12 months of treatment, five (12.5%) were classified as super-responders. No significant differences in baseline characteristics were identified between non-super-responders and super-responders. Super-response rates reported in real-world studies vary considerably according to the definition applied and the characteristics of the study population. In a multicentre real-world study in England, Jackson et al. classified 57.2% of 201 patients with complete 48-week data as super-responders when super-response was defined as the absence of exacerbations and no requirement for maintenance oral corticosteroids [10]. Another benralizumab cohort reported a super-response rate of 63% using the same criteria [33]. These rates may be higher than that observed in our study because we applied the more comprehensive Delphi consensus criteria encompassing multiple clinical domains. Differences in baseline characteristics and previous biologic treatment histories among the cohorts may also have contributed. Because our super-responder group comprised only five patients, comparisons had limited power, and we could not identify baseline factors explaining why some patients achieved super-response.

## 5. Limitations

Several limitations should be acknowledged. The single-centre, retrospective design and modest sample size limit the generalisability of our findings. Following the week 16 assessment, benralizumab was discontinued in 13 clinical non-responders. Consequently, the 12-month analyses were conducted in the 40 patients who continued treatment, and the 12-month findings reflect outcomes in these 40 patients who continued treatment. The small biologic-naïve and super-responder subgroups limited the power of between-group comparisons and precluded reliable identification of predictors of super-response. Previous biologic treatment may have influenced baseline blood eosinophil counts. FeNO measurements were unavailable because they are not routinely performed at our centre; therefore, the relationship between FeNO, atopy, and treatment response could not be evaluated.

## 6. Conclusions

Among patients who continued benralizumab through 12 months, treatment was associated with improvements in asthma control and pulmonary function, marked depletion of peripheral blood eosinophils, and reductions in exacerbations, asthma-related hospitalisations, and systemic corticosteroid courses. No statistically significant differences in improvement were observed among biologic-naïve patients and those switched from omalizumab or mepolizumab. Although older age and atopy were more frequent among week 16 responders, these exploratory findings require confirmation in larger cohorts.

## Figures and Tables

**Table 1:** Baseline demographic, clinical, and functional characteristics of the study population (*n* = 53).

Variable	Overall (*n* = 53)
Age, years	54.68 ± 11.07
Age at asthma onset, years	37.09 ± 12.03
Asthma duration, years	16 (10.5–25)
BMI, kg/m^2^	30 (25.5–36.5)
Female sex, *n* (%)	46 (86.8)
History of smoking, *n* (%)	20 (37.7)
Smoking exposure, pack-years (*n* = 20)	19 (5–27.25)
Presence of rhinitis symptoms, *n* (%)	50 (94.3)
Nasal polyps, *n* (%)	8 (15.1)
NERD, *n* (%)	5 (9.4)
Atopy, *n* (%)	34 (64.2)
GORD, *n* (%)	8 (15.1)
Anxiety/depression, *n* (%)	17 (32.1)
Osteoporosis, *n* (%)	9 (17.0)
CAD, *n* (%)	20 (37.7)
Diabetes mellitus, *n* (%)	10 (18.9)
OSAS, *n* (%)	2 (3.8)
Perennial allergen sensitisation, *n* (%)	33 (62.3)
Seasonal allergen sensitisation, *n* (%)	8 (15.1)
Total IgE at benralizumab initiation, IU/mL	112 (34–313.5)
Blood eosinophil count at benralizumab initiation, cells/µL	329 (71.5–534)
FEV_1_ at benralizumab initiation, L	1.78 ± 0.67
FEV_1_ at benralizumab initiation, % predicted	67.72 ± 21.17
ACT score at benralizumab initiation	13.45 ± 4.12
Number of asthma exacerbations in the preceding 12 months	3 (1.5–4)
Number of asthma-related hospitalisations in the preceding 12 months	0 (0–0)
Number of systemic corticosteroid courses in the preceding 12 months	2 (1–4)
Regular oral corticosteroid use at baseline, *n* (%)	4 (7.5)
**Previous biologic treatment history**	
Biologic-naïve	11 (20.8)
Switched from omalizumab to benralizumab	12 (22.6)
Switched from mepolizumab to benralizumab	30 (56.6)

Data are presented as mean ± standard deviation, median (Q1–Q3), or number (percentage). For binary categorical variables, the number and percentage of patients with the specified characteristic are presented. Smoking exposure was calculated among patients with a history of smoking (*n* = 20). ACT, Asthma Control Test; BMI, body mass index; CAD, coronary artery disease; FEV_1_, forced expiratory volume in one second; GORD, gastro-oesophageal reflux disease; IgE, immunoglobulin E; NERD, non-steroidal anti-inflammatory drug-exacerbated respiratory disease; OSAS, obstructive sleep apnoea syndrome. Q1 and Q3 denote the 25th and 75th percentiles, respectively.

**Table 2:** Changes in clinical, laboratory, and functional outcomes during benralizumab treatment. (**A**) Changes in clinical events among patients who continued treatment for 12 months. (**B**) Changes in laboratory findings, asthma control, and pulmonary function.

**(A)**								
**Variable**		**12 Months Before Treatment (*n* = 40)**			**First 12 Months of Benralizumab Treatment (*n* = 40)**	***p* Value**		**Effect Size**
Number of asthma exacerbations		3 (2–4)			0 (0–1)	**<0.001**		**0.808 ^b^**
Number of asthma-related hospitalisations		0 (0–0)			0 (0–0)	**0.015**		**0.384 ^b^**
Patients with ≥1 asthma-related hospitalisation, *n* (%)		7 (17.5)			1 (2.5)	—		—
Number of systemic corticosteroid courses		2 (1–3)			0 (0–0)	**<0.001**		**0.746 ^b^**
**(B)**								
**Variable**	**Baseline (*n* = 53)**	**Week 16 (*n* = 53)**	***p* Value**	**Effect Size**	**Baseline (*n* = 40)**	**Month 12 (*n* = 40)**	***p* Value**	**Effect Size**
Peripheral blood eosinophil count, cells/µL	329 (71.5–534)	1 (0–4)	**<0.001**	**0.861 ^b^**	288.5 (64.75–510.75)	2.5 (1–4)	**<0.001**	**0.797 ^b^**
ACT score	13.45 ± 4.12	17.26 ± 4.87	**<0.001**	**0.828 ^a^**	13.83 ± 4.22	21.30 ± 3.39	**<0.001**	**1.890 ^a^**
FEV_1_, L	1.78 ± 0.67	1.98 ± 0.73	**<0.001**	**0.326 ^a^**	1.68 ± 0.65	1.98 ± 0.65	**<0.001**	**0.799 ^a^**
FEV_1_, % predicted	67.72 ± 21.17	75.19 ± 23.18	**<0.001**	**0.576 ^a^**	67.98 ± 21.77	78.58 ± 23.39	**<0.001**	**0.713 ^a^**

Effect size: ^a^, Cohen’s d; ^b^, rank-biserial correlation coefficient. The direction of change is indicated by the baseline and follow-up values. Data are presented as mean ± standard deviation, median (Q1–Q3), or number (percentage). ACT, Asthma Control Test; FEV_1_, forced expiratory volume in one second. The baseline-to-week 16 comparisons included 53 patients with a week 16 assessment, whereas the baseline-to-month 12 comparisons included 40 patients who continued benralizumab treatment for 12 months. Clinical event counts were evaluated by comparing the 12 months before treatment with the first 12 months of benralizumab treatment. Statistically significant *p* values are shown in bold. Q1 and Q3 denote the 25th and 75th percentiles, respectively.

**Table 3:** Comparison of baseline characteristics according to treatment response at week 16.

Variable	Non-Responders (*n* = 13)	Responders (*n* = 40)	*p* Value
Age, years	47.62 ± 11.13	56.98 ± 10.16	**0.007**
Age at asthma onset, years	32.69 ± 12.93	38.53 ± 11.53	0.130
Asthma duration, years	12 (8–26)	17 (11–24.75)	0.402
BMI, kg/m^2^	30 (25.7–36.5)	30 (25.25–36.75)	0.733
Female sex, *n* (%)	9 (69.2)	37 (92.5)	0.053
History of smoking, *n* (%)	5 (38.5)	15 (37.5)	1.000
Smoking exposure, pack-years	0 (0–13.5)	0 (0–8.75)	0.896
Presence of rhinitis symptoms, *n* (%)	11 (84.6)	39 (97.5)	0.145
Nasal polyps, *n* (%)	1 (7.7)	7 (17.5)	0.662
GORD, *n* (%)	2 (15.4)	6 (15.0)	1.000
Anxiety/depression, *n* (%)	5 (38.5)	12 (30.0)	0.734
Osteoporosis, *n* (%)	0 (0)	9 (22.5)	0.092
CAD, *n* (%)	4 (30.8)	16 (40.0)	0.744
Diabetes mellitus, *n* (%)	3 (23.1)	7 (17.5)	0.692
OSAS, *n* (%)	1 (7.7)	1 (2.5)	0.434
NERD, *n* (%)	2 (15.4)	3 (7.5)	0.586
Atopy, *n* (%)	5 (38.5)	29 (72.5)	**0.044**
Perennial allergen sensitisation, *n* (%)	5 (38.5)	28 (70.0)	0.054
Seasonal allergen sensitisation, *n* (%)	2 (15.4)	6 (15.0)	1.000
Total IgE at benralizumab initiation, IU/mL	106 (23–406)	148.5 (38.25–283)	0.756
Blood eosinophil count at benralizumab initiation, cells/µL	332 (66.5–637.5)	288.5 (64.75–510.75)	0.934
FEV_1_ at benralizumab initiation, L	2.09 ± 0.63	1.68 ± 0.65	0.051
FEV_1_ at benralizumab initiation, % predicted	66.92 ± 20.01	67.98 ± 21.77	0.878
ACT score at benralizumab initiation	12.31 ± 3.71	13.83 ± 4.22	0.253
Number of asthma exacerbations in the preceding 12 months	2 (1–4.5)	3 (2–4)	0.745
Number of asthma-related hospitalisations in the preceding 12 months	0 (0–1)	0 (0–0)	0.413
Number of systemic corticosteroid courses in the preceding 12 months	2 (1–4)	2 (1–3)	0.343
Regular oral corticosteroid use at baseline, *n* (%)	0 (0)	4 (10.0)	0.561
**Previous biologic treatment, *n* (%)**			
Biologic-naïve	3 (23.1)	8 (20.0)	0.625
Switched from omalizumab to benralizumab	4 (30.8)	8 (20.0)	
Switched from mepolizumab to benralizumab	6 (46.2)	24 (60.0)	

Data are presented as mean ± standard deviation, median (Q1–Q3), or number (percentage). ACT, Asthma Control Test; BMI, body mass index; CAD, coronary artery disease; FEV_1_, forced expiratory volume in one second; GORD, gastro-oesophageal reflux disease; IgE, immunoglobulin E; NERD, non-steroidal anti-inflammatory drug-exacerbated respiratory disease; OSAS, obstructive sleep apnoea syndrome. Smoking exposure was calculated for the entire group, with never-smokers coded as zero. Statistically significant *p* values are shown in bold. Q1 and Q3 denote the 25th and 75th percentiles, respectively.

**Table 4:** Comparison of baseline characteristics according to response level at month 12 of benralizumab treatment.

Variable	Non-Super-Responders (*n* = 35)	Super-Responders (*n* = 5)	*p* Value
Age, years	58 (48–66)	50 (49–62.5)	0.634
Age at asthma onset, years	39.20 ± 11.86	33.80 ± 8.26	0.334
Asthma duration, years	17 (10–25)	20 (13.5–28.5)	0.436
BMI, kg/m^2^	30 (25–37)	30 (28.5–33.9)	0.905
Female sex, *n* (%)	32 (91.4)	5 (100)	1.000
History of smoking, *n* (%)	13 (37.1)	2 (40.0)	1.000
Smoking exposure, pack-years	0 (0–20)	0 (0–3.5)	0.781
Presence of rhinitis symptoms, *n* (%)	34 (97.1)	5 (100)	1.000
Nasal polyps, *n* (%)	7 (20.0)	0 (0)	0.565
GORD, *n* (%)	5 (14.3)	1 (20.0)	1.000
Anxiety/depression, *n* (%)	10 (28.6)	2 (40.0)	0.627
Osteoporosis, *n* (%)	8 (22.9)	1 (20.0)	1.000
CAD, *n* (%)	13 (37.1)	3 (60.0)	0.373
Diabetes mellitus, *n* (%)	7 (20.0)	0 (0)	0.565
OSAS, *n* (%)	1 (2.9)	0 (0)	1.000
NERD, *n* (%)	3 (8.6)	0 (0)	1.000
Atopy, *n* (%)	25 (71.4)	4 (80.0)	1.000
Perennial allergen sensitisation, *n* (%)	24 (68.6)	4 (80.0)	1.000
Seasonal allergen sensitisation, *n* (%)	5 (14.3)	1 (20.0)	1.000
Total IgE at benralizumab initiation, IU/mL	157 (34–297)	102 (49.5–281)	0.874
Blood eosinophil count at benralizumab initiation, cells/µL	226 (55–432)	527 (295–971.5)	0.133
FEV_1_ at benralizumab initiation, L	1.69 ± 0.69	1.62 ± 0.42	0.829
FEV_1_ at benralizumab initiation, % predicted	67.71 ± 23.24	69.80 ± 4.76	0.644
ACT score at benralizumab initiation	13.74 ± 4.26	14.40 ± 4.39	0.749
Number of asthma exacerbations in the preceding 12 months	3 (1–4)	3 (2–4)	0.905
Number of asthma-related hospitalisations in the preceding 12 months	0 (0–0)	0 (0–0)	0.293
Number of systemic corticosteroid courses in the preceding 12 months	2 (0.75–4)	3 (0.5–3)	0.919
Regular oral corticosteroid use at baseline, *n* (%)	4 (11.4)	0 (0)	1.000
**Previous biologic treatment, *n* (%)**			
Biologic-naïve	6 (17.1)	2 (40.0)	0.120
Switched from omalizumab to benralizumab	6 (17.1)	2 (40.0)	
Switched from mepolizumab to benralizumab	23 (65.7)	1 (20.0)	

Data are presented as mean ± standard deviation, median (Q1–Q3), or number (percentage). ACT, Asthma Control Test; BMI, body mass index; CAD, coronary artery disease; FEV_1_, forced expiratory volume in one second; GORD, gastro-oesophageal reflux disease; IgE, immunoglobulin E; NERD, non-steroidal anti-inflammatory drug-exacerbated respiratory disease; OSAS, obstructive sleep apnoea syndrome. Smoking exposure was calculated for the entire group, with never-smokers coded as zero. Q1 and Q3 denote the 25th and 75th percentiles, respectively.

**Table 5:** Comparison of changes in clinical, laboratory, and functional outcomes during benralizumab treatment according to previous biologic treatment history. (**A**) Changes at week 16. (**B**) Changes at month 12.

**(A)**				
**Variable**	**Biologic-Naïve** **(*n* = 11)**	**Switched from** **Omalizumab (*n* = 12)**	**Switched from** **Mepolizumab (*n* = 30)**	***p* Value**
ACT score, change from baseline	4.64 ± 3.70	2.92 ± 5.25	3.87 ± 4.72	0.675
Blood eosinophil count, cells/µL, change from baseline	−464 (−1080 to −136)	−406 (−538 to −187.75)	−133.5 (−438.75 to −41)	0.118
FEV_1_, L, change from baseline	0.30 (0.1–0.51)	0.15 (−0.17 to 0.64)	0.10 (−0.01 to 0.42)	0.530
FEV_1_, % predicted, change from baseline	10 (1–17)	4.5 (−6 to 19.5)	2 (−0.25 to 15.25)	0.521
**(B)**				
**Variable**	**Biologic-Naïve** **(*n* = 8)**	**Switched from** **Omalizumab (*n* = 8)**	**Switched from** **Mepolizumab (*n* = 24)**	***p* Value**
Annual number of asthma exacerbations, change from baseline	−3 (−5.25 to −1)	−2 (−4.75 to −2)	−2 (−3.75 to −1)	0.402
Annual number of asthma-related hospitalisations, change from baseline	0 (−0.75 to 0)	0 (0–0)	0 (0–0)	0.717
Annual number of systemic corticosteroid courses, change from baseline	−2 (−3 to −1)	−2 (−3 to −0.25)	−1.5 (−3 to 0)	0.502
ACT score, change from baseline	7.38 ± 3.11	6.38 ± 4.84	7.88 ± 3.98	0.659
Blood eosinophil count, cells/µL, change from baseline	−465.5 (−1889 to −91.75)	−405 (−852.25 to −245)	−136.5 (−429.25 to −41.5)	0.224
FEV_1_, L, change from baseline	0.49 ± 0.42	0.38 ± 0.35	0.21 ± 0.35	0.134
FEV_1_, % predicted, change from baseline	17.88 ± 19.40	11.13 ± 12.88	8.00 ± 13.57	0.271

Data are presented as mean ± standard deviation or median (Q1–Q3). Change was calculated as the follow-up value minus the baseline value. ACT, Asthma Control Test; FEV_1_, forced expiratory volume in one second. Q1 and Q3 denote the 25th and 75th percentiles, respectively.

## Data Availability

The data are available from the corresponding author upon reasonable request due to ethical restrictions and data protection considerations.

## References

[1] Global Initiative for Asthma (2026). Global Strategy for Asthma Management and Prevention. https://ginasthma.org/2026-gina-strategy-report/.

[2] Hekking P.W., Wener R.R., Amelink M., Zwinderman A.H., Bouvy M.L., Bel E.H. (2015). The prevalence of severe refractory asthma. J. Allergy Clin. Immunol..

[3] Kolbeck R., Kozhich A., Koike M., Peng L., Andersson C.K., Damschroder M.M., Reed J.L., Woods R., Dall’Acqua W.W., Stephens G.L. (2010). MEDI-563, a humanized anti-IL-5 receptor α mAb with enhanced antibody-dependent cell-mediated cytotoxicity function. J. Allergy Clin. Immunol..

[4] FitzGerald J.M., Bleecker E.R., Nair P., Korn S., Ohta K., Lommatzsch M., Ferguson G.T., Busse W.W., Barker P., Sproule S. (2016). Benralizumab, an anti-interleukin-5 receptor α monoclonal antibody, as add-on treatment for patients with severe, uncontrolled, eosinophilic asthma (CALIMA): A randomised, double-blind, placebo-controlled phase 3 trial. Lancet.

[5] Bleecker E.R., FitzGerald J.M., Chanez P., Papi A., Weinstein S.F., Barker P., Sproule S., Gilmartin G., Aurivillius M., Werkström V. (2016). Efficacy and safety of benralizumab for patients with severe asthma uncontrolled with high-dosage inhaled corticosteroids and long-acting β2-agonists (SIROCCO): A randomised, multicentre, placebo-controlled phase 3 trial. Lancet.

[6] Nair P., Wenzel S., Rabe K.F., Bourdin A., Lugogo N.L., Kuna P., Barker P., Sproule S., Ponnarambil S., Goldman M. (2017). Oral glucocorticoid-sparing effect of benralizumab in severe asthma. N. Engl. J. Med..

[7] Korn S., Bourdin A., Chupp G., Cosio B.G., Arbetter D., Shah M., Garcia Gil E. (2021). Integrated safety and efficacy among patients receiving benralizumab for up to 5 years. J. Allergy Clin. Immunol. Pract..

[8] Martínez-Moragón E., García-Moguel I., Nuevo J., Resler G., ORBE Study Investigators (2021). Real-world study in severe eosinophilic asthma patients refractory to anti-IL5 biological agents treated with benralizumab in Spain (ORBE study). BMC Pulm. Med..

[9] Schleich F., Moermans C., Seidel L., Kempeneers C., Louis G., Rogister F., Tombu S., Pottier L., Poirrier A.-L., Ziant S. (2023). Benralizumab in severe eosinophilic asthma in real life: Confirmed effectiveness and contrasted effect on sputum eosinophilia versus exhaled nitric oxide fraction—PROMISE. ERJ Open Res..

[10] Jackson D.J., Burhan H., Menzies-Gow A., Pfeffer P., Nanzer A., Garcia Gil E., Morris T., Tran T.N., Hirsch I., Dube S. (2022). Benralizumab effectiveness in severe asthma is independent of previous biologic use. J. Allergy Clin. Immunol. Pract..

[11] Yamaguchi M., Nishimura Y., Takumi Y., Hayashi N., Sakamoto K., Tohda Y. (2024). Real-world safety and effectiveness of benralizumab in Japanese patients with severe asthma: A multicenter prospective observational study. J. Asthma Allergy.

[12] Çelebi Sözener Z., Özdel Öztürk B., Şahinoğlu E., Köylüce S., Türk M., Aydın Ö., Sin B.A., Mungan D., Yılmaz I., Bavbek S. (2025). Benralizumab in severe eosinophilic asthma: Real-life data from Türkiye. Respir. Med..

[13] Özden Ş., Tepetam F.M., Bozkurt Y., Terzioğlu Şahin F., Aynacı A., Avcı H.F., Dursun M., Demirkol M.A., Yıldırım N. (2026). Real-world effectiveness of benralizumab in severe eosinophilic asthma: A 32-week evaluation. Int. Arch. Allergy Immunol..

[14] Graham B.L., Steenbruggen I., Miller M.R., Barjaktarevic I.Z., Cooper B.G., Hall G.L., Hallstrand T.S., Kaminsky D.A., McCarthy K., McCormack M.C. (2019). Standardization of Spirometry 2019 Update: An official American Thoracic Society and European Respiratory Society technical statement. Am. J. Respir. Crit. Care Med..

[15] Upham J.W., Le Lievre C., Jackson D.J., Masoli M., Wechsler M.E., Price D.B., Mansur A., Detoraki A., Altraja A., James A. (2021). Defining a severe asthma super-responder: Findings from a Delphi process. J. Allergy Clin. Immunol. Pract..

[16] Jackson D.J., Pelaia G., Emmanuel B., Tran T.N., Cohen D., Shih V.H., Shavit A., Arbetter D., Katial R., Rabe A.P.J. (2024). Benralizumab in severe eosinophilic asthma by previous biologic use and key clinical subgroups: Real-world XALOC-1 programme. Eur. Respir. J..

[17] Mihaltan F., Csoma Z., Pauk N., Irás B., Baukienė J., Teodorescu G., Malakauskas K., Staevska M. (2025). Benralizumab outcomes in patients with severe eosinophilic asthma treated in real-life settings: Results of the BREEZE study in 5 countries from Central Eastern Europe and Baltics. J. Asthma Allergy.

[18] Schatz M., Kosinski M., Yarlas A.S., Hanlon J., Watson M.E., Jhingran P. (2009). The minimally important difference of the Asthma Control Test. J. Allergy Clin. Immunol..

[19] Padilla-Galo A., Moya Carmona I., Ausín P., Carazo Fernández L., García-Moguel I., Velasco-Garrido J.L., Andújar-Espinosa R., Casas-Maldonado F., Martínez-Moragón E., Rivera C.M. (2023). Achieving clinical outcomes with benralizumab in severe eosinophilic asthma patients in a real-world setting: ORBE II study. Respir. Res..

[20] Sandhu Y., Harada N., Sasano H., Harada S., Ueda S., Takeshige T., Tanabe Y., Ishimori A., Matsuno K., Abe S. (2023). Pretreatment frequency of circulating Th17 cells and FeNO levels predicted the real-world response after 1 year of benralizumab treatment in patients with severe asthma. Biomolecules.

[21] Numata T., Araya J., Okuda K., Miyagawa H., Minagawa S., Ishikawa T., Hara H., Kuwano K. (2022). Long-term efficacy and clinical remission after benralizumab treatment in patients with severe eosinophilic asthma: A retrospective study. J. Asthma Allergy.

[22] Cameli P., Aliani M., Altieri E., Bracciale P., Brussino L., Caiaffa M.F., Canonica G.W., Caruso C., Centanni S., D’Amato M. (2024). Sustained effectiveness of benralizumab in naïve and biologics-experienced severe eosinophilic asthma patients: Results from the ANANKE study. J. Asthma Allergy.

[23] Pelaia C., Crimi C., Benfante A., Caiaffa M.F., Calabrese C., Carpagnano G.E., Ciotta D., D’Amato M., Macchia L., Nolasco S. (2021). Therapeutic effects of benralizumab assessed in patients with severe eosinophilic asthma: Real-life evaluation correlated with allergic and nonallergic phenotype expression. J. Asthma Allergy.

[24] Pauk N., Valena T., Krčmová I., Heribanová L., Hutyrová B., Mokošová R., Voříšek M., Maruščák M. (2026). Real-world clinical experience with benralizumab for severe eosinophilic asthma in the Czech Republic: Fewer corticosteroids and better symptom control. J. Asthma.

[25] Rattu A., Dixey P., Charles D., Brightling C., Chung K.F., Bossios A., Bourdin A., Djukanovic R., Dahlén S., Fleming L. (2026). Predictors of response to biologics for severe asthma: A systematic review and meta-analysis. Allergy.

[26] Sposato B., Ricci A., Camiciottoli G., Carpagnano G.E., Pelaia C., Santus P., Pelaia G., Palmiero G., Di Tomassi M., Ronchi M.C. (2023). Influence of allergic status and nasal polyposis on long-term benralizumab response in eosinophilic severe asthma. Clin. Ter..

[27] Chipps B.E., Newbold P., Hirsch I., Trudo F., Goldman M. (2018). Benralizumab efficacy by atopy status and serum immunoglobulin E for patients with severe, uncontrolled asthma. Ann. Allergy Asthma Immunol..

[28] Di Bona D., Crimi C., D’Uggento A.M., Benfante A., Caiaffa M.F., Calabrese C., Campisi R., Carpagnano G.E., Ciotta D., D’Amato M. (2022). Effectiveness of benralizumab in severe eosinophilic asthma: Distinct sub-phenotypes of response identified by cluster analysis. Clin. Exp. Allergy.

[29] Pelaia C., Crimi C., Nolasco S., Carpagnano G.E., Brancaccio R., Buonamico E., Campisi R., Gagliani C., Patella V., Pelaia G. (2021). Switch from omalizumab to benralizumab in allergic patients with severe eosinophilic asthma: A real-life experience from Southern Italy. Biomedicines.

[30] Drick N., Milger K., Seeliger B., Fuge J., Korn S., Buhl R., Schuhmann M., Herth F., Kendziora B., Behr J. (2020). Switch from IL-5 to IL-5-receptor α antibody treatment in severe eosinophilic asthma. J. Asthma Allergy.

[31] Matucci A., Maggi E., Vultaggio A. (2019). Eosinophils, the IL-5/IL-5Rα axis, and the biologic effects of benralizumab in severe asthma. Respir. Med..

[32] Caruso C., Cameli P., Altieri E., Aliani M., Bracciale P., Brussino L., Caiaffa M.F., Canonica G.W., Centanni S., D’Amato M. (2022). Switching from one biologic to benralizumab in patients with severe eosinophilic asthma: An ANANKE study post hoc analysis. Front. Med..

[33] Miralles-López J.C., Andújar-Espinosa R., Bravo-Gutiérrez F.J., Castilla-Martínez M., Flores-Martín I., Alemany-Francés M.L., Pajarón-Fernández M.J., Mora-González A., Cabrejos-Perotti S., El-Molaka Z. (2022). Analysis of response of severe eosinophilic asthmatic patients to benralizumab. Allergol. Immunopathol..

